# Mortality Outcomes and Contributing Risk Factors in Patients with Hospital-Associated Disability

**DOI:** 10.3390/jcm13164798

**Published:** 2024-08-15

**Authors:** Soo-Jeong Jo, So-Hee Lee, Hyo-Jin Min, Hee-Ji Kim, Hyun-Ho Kong

**Affiliations:** 1 Department of Rehabilitation Medicine, Chungbuk National University Hospital, Cheongju 28644, Republic of Korea; tnwjd605777@gmail.com (S.-J.J.); spdlqj7611@naver.com (S.-H.L.); 2 Department of Clinical Pharmacology and Therapeutics, Chungbuk National University Hospital, Cheongju 28644, Republic of Korea; hj_min@cbnuhctc.com; 3 Department of Medicine, Chungbuk National University Graduate School, Cheongju 28644, Republic of Korea; 4 Department of Nursing, Chungbuk National University Hospital, Cheongju 28644, Republic of Korea; sskimheejiss@naver.com; 5 Department of Rehabilitation Medicine, Chungbuk National University College of Medicine, Cheongju 28644, Republic of Korea

**Keywords:** activities of daily living, hospitalization, mortality, risk factors, anthropometry

## Abstract

**Background/Objectives**: Hospital-associated disability (HAD), a functional decline following acute hospitalization, is a common complication associated with mortality and unfavorable prognoses in patients admitted to acute care hospitals. However, few studies have investigated mortality and associated factors in patients with HAD and have been limited by inconsistent HAD assessment tools and criteria. This study investigated mortality and risk factors in patients with HAD using specific criteria. **Methods**: This retrospective study evaluated patients referred to the Department of Rehabilitation Medicine with suspected HAD between June 2022 and March 2023. The collected data included medical histories, diagnostic tests for HAD (including muscle strength, balance, and modified Barthel Index), and bioelectrical impedance analysis (BIA). Multivariate logistic regression analysis was conducted to identify factors associated with mortality. Kaplan–Meier survival curves were constructed for mortality at 3 and 7 months. **Results**: A total of 455 patients were identified, among which 206 patients diagnosed with HAD (73.1 ± 12.5 years) were included in the analysis. The 3-month mortality rate was 27.2%. In the multivariate analysis, male sex (odds ratio (OR), 3.23; *p* < 0.01), a history of cancer (OR, 2.18; *p* < 0.05), and a low phase angle (OR, 0.69; *p* < 0.05) were significantly associated with mortality. A phase angle < 2.9° on BIA was associated with a significant increase in 3-month (hazard ratio (HR), 1.85; 95% confidence interval (CI), 1.06–3.23) and 7-month (HR, 2.80; 95% CI, 1.75–4.98) mortality. **Conclusions**: Patients with HAD had a high mortality rate, with several factors, particularly low BIA phase angles, associated with increased mortality.

## 1. Introduction

Extensive research has been conducted on the risk of developing functional decline in hospitalized patients with acute health problems, affecting approximately 30% of patients over the age of 70 years [1]. This condition, commonly known as hospital-associated disability (HAD), is characterized by a loss of independence in activities of daily living (ADLs) following hospitalization owing to acute medical illnesses [2,3]. This decline in ADLs manifests as limitations in performing daily tasks, including bathing, dressing, eating, and urinating, as well as mobility functions such as standing up from a chair or bed and walking [2]. The deterioration in ADLs may be attributed directly to the underlying cause of hospitalization or to secondary effects associated with acute hospitalization, such as immobility, polypharmacy, and undernutrition [2,4].

HAD can adversely affect clinical outcomes and result in an increased utilization of medical resources. When patients experience a decline in ADLs, they become incapable of living independently, resulting in higher admission rates to healthcare facilities, longer hospital stays, increased readmission rates, and elevated short- and long-term mortality rates [5,6,7,8]. Furthermore, an increase in the number of patients with HAD leads to higher medical expenditures in socioeconomic terms and increased utilization of other resources, such as caregiver services [9]. Approximately half of the newly occurring loss in ADLs is attributed to hospital admissions, highlighting the importance of HAD for doctors, patients, and their carers [10].

Previous studies on HAD mainly focused on its prevalence [3,11], risk factors [10,12,13,14], and functional outcomes [6,15]. However, few studies have examined the mortality rate of patients with HAD [2,3], and most of these studies had limitations, as functional assessment was mainly conducted through self-report methods rather than objective and quantitative physical function tests [6,9,12,15,16]. In addition, while there have been reports on the associations between body composition analyses (such as the skeletal muscle index, percentage body fat, bioelectrical impedance analysis (BIA), and phase angle) and outcomes in critically ill patients or those with a history of cancer [17,18,19,20,21], no studies have reported these associations in patients with HAD.

Therefore, this study aimed to investigate the mortality rate of HAD and its associated risk factors using various objective and quantitative assessment tools.

## 2. Materials and Methods

### 2.1. Study Population

This single-center retrospective study assessed patients admitted to a tertiary hospital between June 2022 and March 2023. Participants included those referred for consultation to the Department of Rehabilitation Medicine, with suspicion of ‘disuse syndrome’ (Korea Rehabilitation Impairment Category (KRIC) 22). ‘Disuse syndrome’ refers to the condition of functional decline after acute hospitalization, despite patients having been previously independent in their ADLs, and this term is indicated as ‘HAD’ in this study.

**Figure 1:**
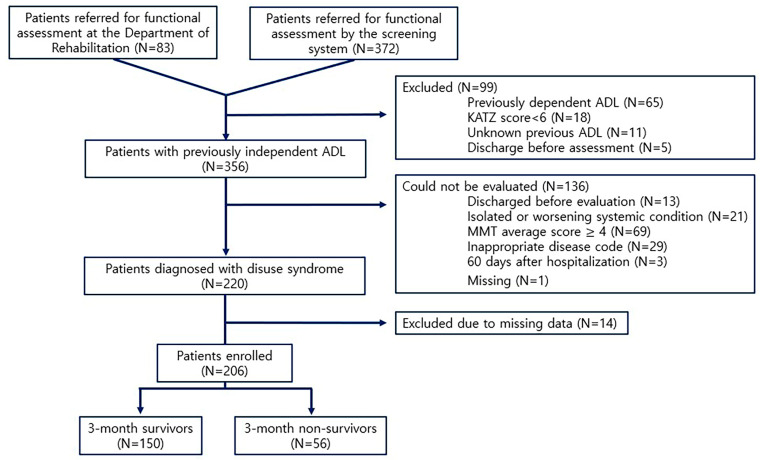
Study flow chart. MMT, manual muscle test; ADL, activity of daily living.

The inclusion criteria were as follows: (i) patients referred for a diagnosis of HAD through the screening system of the Hospital Information System (HIS) 14 days after hospitalization (n = 372), and (ii) those referred directly to the Department of Rehabilitation Medicine for a diagnosis of HAD (n = 83). The exclusion criteria were as follows: (i) those who failed to meet the diagnostic criteria of ‘disuse syndrome (KRIC 22)’ (n = 195), (ii) those whose general condition deteriorated or who were discharged before evaluation and failed to undergo tests (n = 39), and (iii) those with insufficient medical records (n = 15). As a result, a total of 206 patients (115 males and 91 females) diagnosed with HAD who met the aforementioned inclusion and exclusion criteria were included in the analysis (Figure 1).

Data on age, sex, anthropometric factors (height, weight, body mass index (BMI)), underlying diseases (coronary artery disease, congestive heart failure, cerebrovascular disease, chronic obstructive pulmonary disease (COPD), diabetes mellitus, chronic kidney disease, hypertension, history of cancer), and smoking history were collected. In addition, information related to the patient’s hospitalization, such as length of stay (LOS), intensive care unit (ICU) admission, length of ICU stay, diagnostic code, and department requesting the consultation, was obtained. Information on whether patients died was investigated at an average of 3 and 7 months after the diagnosis of HAD through hospital records or by contacting patients’ family members. Data collection for the patients was carried out by a specialized nurse. Ethical approval was obtained from the Institutional Review Board (IRB) of the tertiary hospital (IRB number: 2023-06-032).

### 2.2. Automated Screening System for Hospital-Associated Disability

**Figure 2:**
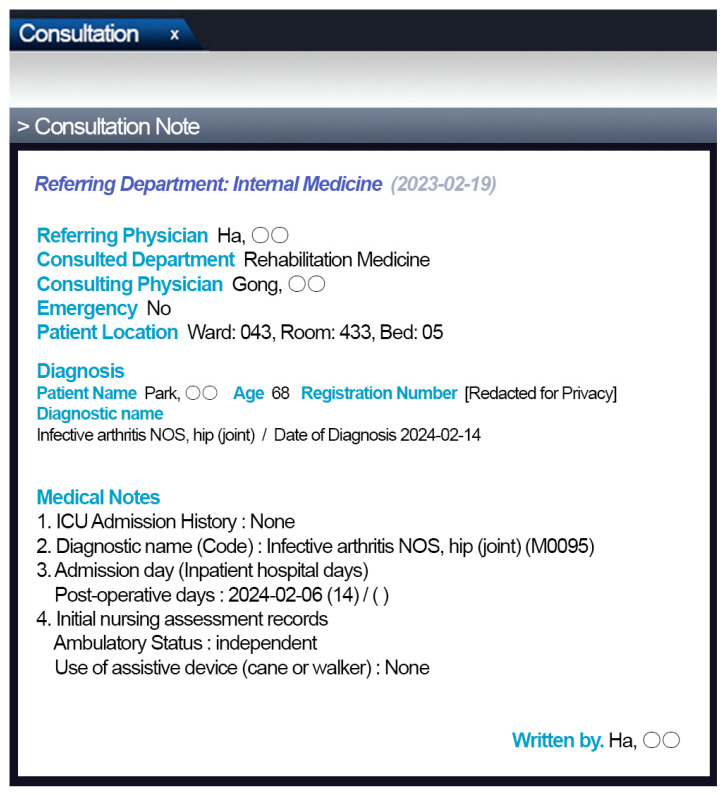
HAD screening system. HAD, hospital-associated disability.

We developed a system to automatically screen individuals at risk of developing HAD using patient information from the HIS (Figure 2). This system automatically refers patients who have been hospitalized for more than 2 weeks but less than 60 days to the Department of Rehabilitation Medicine. However, patients with conditions such as strokes, lower limb fractures, and spinal cord injuries that do not satisfy the KRIC 22 criteria were automatically excluded from the screening. Once referred, a rehabilitation medicine doctor evaluated their previous independent ADLs and then performed physical function tests. If the diagnostic criteria for HAD were met based on these tests, the patient was diagnosed with HAD.

### 2.3. Diagnostic Criteria for Hospital-Associated Disability

In this study, functional decline was characterized by weakness in muscle strength as well as deterioration in balance ability or ADLs based on the criteria of ‘disuse syndrome (KRIC 22)’. The diagnostic criteria for ‘disuse syndrome’ followed those established by the Health Insurance Review & Assessment Service, Republic of Korea [22]. As previously mentioned, we defined ‘disuse syndrome’ as equivalent to HAD in our study. A rehabilitation medicine doctor diagnosed HAD through a medical examination and functional evaluation. If the pre-admission ADL function was confirmed to be independent using the Katz ADL score acquired from the patient or caregiver, a functional assessment was conducted [23]. Functional evaluation consisted of two physical function tests, namely Manual Muscle Testing (MMT) and the Berg Balance Scale (BBS), as well as the Modified Barthel Index (MBI). All functional evaluations were conducted by an experienced physical therapist. HAD was diagnosed if the sum of the muscle strength scores was less than 48 points, with a concomitant BBS score of <40 points or an MBI score of <80 points [24,25].

### 2.4. Quantitative Assessment of Functional Decline

The MMT scores were measured for each of the three muscles of the upper and lower extremities (shoulder joint abductor, elbow joint flexor, wrist joint extensor, hip joint flexor, knee joint extensor, and ankle joint extensor (plantar flexor)). Muscle strength was evaluated using the Medical Research Council (MRC) score, which assesses the strength of each muscle on a scale of 0 to 5 points, resulting in a total score of 0 to 60 points [24].

The BBS is a clinical tool used to evaluate balance ability and the risk of falling. It comprises 14 items, including sitting and standing up, standing without assistance, sitting without assistance, and standing with one’s eyes closed. Each item is measured on a scale from 0 to 4, where 0 represents a state in which the test may not be performed, and 4 represents a state in which the test may be performed independently. The total score of the BBS is 56 points, with higher scores indicating better balance ability closer to the norm [26].

The MBI is a standardized tool used to assess the degree of dependence in ADLs and consists of 10 items. The test was conducted by an experienced physical therapist in the tertiary hospital. Each item is rated with 5 grades according to the degree of assistance needed by the patient, with lower scores indicating greater levels of dependence. The maximum score is 100, which represents complete independence in ADLs [27].

Hand grip strength in the dominant hand was measured using a digital dynamometer (T.K.5710, grip-D Takei, Tokyo, Japan (Takei Scientific Instruments Co., Ltd.)). The patient was positioned in a sitting position with their elbow flexed at 90°, and hand grip strength was measured with the wrist and forearm in a neutral position [28,29]. In instances where the patient was unable to sit, the test was conducted in the supine position. Hand grip strength was measured twice, and the higher measurement value was recorded [30]. Owing to the significant difference in normal handgrip strength values between males and females, patients were divided into the low-handgrip-strength and standard groups according to the criteria of the Asian Working Group for Sarcopenia (AWGS) 2019 (normal handgrip strength values: ≥28.0 kg for males and ≥18.0 kg for females) [30].

### 2.5. Measurement of Body Composition Using Bioelectrical Impedance Analysis

Body composition was evaluated using a BIA device (the BWA 2.0^®^, InBody Corp., Seoul, Republic of Korea). The BWA 2.0 body composition analyzer employs clamp-type electrodes, with one electrode on each wrist and one on each ankle. The BIA assessment was conducted by an experienced physical therapist within 3 days following the consultation request for the diagnosis of HAD. The BIA provides measurements for the whole-body muscle and fat weights, the whole-body phase angle (PhA) at 50 kHz, the lean body mass, percent body fat (PBF), and the skeletal muscle mass index (SMI). Owing to the significant difference in normal SMI values between males and females, patients were divided into the low-muscle-mass and standard groups according to the BIA criteria of the Asian Working Group for Sarcopenia (AWGS) 2019 (normal SMI values: ≥7.0 kg/m^2^ for males and ≥5.7 kg/m^2^ for females) [30]. BIA measures the body resistance (R) and reactance (Xc) by recording a voltage drop in applied current. Resistance is related to total body tissue fluid, while reactance is associated with conserving cell membranes [17]. Reactance leads to a delay in the current relative to the voltage, resulting in a phase shift. This shift can be geometrically described as the angular transformation derived from the ratio of reactance to resistance, known as the PhA [31]. Low PhAs indicate cell death or reduced cell integrity, whereas high PhAs indicate a greater amount of intact cell membranes [32].

### 2.6. Statistical Analysis

Continuous variables were expressed as means and standard deviations, and categorical variables are expressed as numbers and percentages. To compare characteristics between 3-month survivors and non-survivors among patients with HAD, Student’s *t*-test and the chi-squared test were employed for continuous and categorical variables, respectively. Univariate logistic regression analysis was performed to identify risk factors related to mortality, and variables with *p*-values less than 0.05 were considered statistically significant. Multivariate logistic regression analysis was performed using the stepwise elimination approach, including statistically significant variables from the univariate analysis. In addition, receiver operating characteristic (ROC) curve analysis using the Youden method was conducted to determine the optimal cut-off value for the PhA. The Kaplan–Meier method was used to determine whether the mortality rate according to the PhA cut-off was significantly different at the 3- and 7-month time points. Differences in survival curves were assessed using the log-rank test. All statistical analyses were performed using SPSS version 25.0 (IBM Corporation, Chicago, IL, USA), SAS^®^ analytics Pro Version 9.4 (SAS Institute Inc., Cary, NC, USA), and MedCalc^®^ Statistical Software version 20.009 (MedCalc Software Ltd., Ostend, Belgium).

## 3. Results

### 3.1. Patient Characteristics

The characteristics of the patients with HAD are shown in Table 1. Of the 206 patients analyzed, 56 (27.1%) died within the 3-month follow-up period and 79 (38.8%) died within the average 7-month follow-up period after diagnosis of HAD. In a comparison between survivors and non-survivors 3 months after HAD diagnosis, there were statistically significant differences between the two groups in terms of sex (male, *p* < 0.001), history of cancer (*p* < 0.01), PhA (3.5 ± 1.2° in survivors vs. 3.1 ± 1.1° in non-survivors; *p* < 0.05), and PBF (29.1 ± 10.9% in survivors vs. 25.4 ± 11.4% in non-survivors; *p* < 0.05). However, no statistically significant differences were observed in age, BMI, ICU admission, or underlying diseases, excluding cancer history.

### 3.2. Risk Factors Associated with 3-Month Mortality 

Table 2 presents the risk factors affecting the 3-month mortality in patients with HAD. In the univariate analysis, sex (male, odds ratio (OR), 3.16; 95% confidence interval (CI), 1.60–6.27; *p* < 0.01), history of cancer (OR, 2.59; 95% CI, 1.38–4.86; *p* < 0.01), low PhA (OR, 0.74; 95% CI, 0.54–0.99; *p* < 0.05), and PBF (OR, 0.97; 95% CI, 0.94–0.99; *p* < 0.05) were associated with the mortality within 3 months after diagnosis of HAD. In the multivariate logistic regression analysis, male sex (OR, 3.23; 95% CI, 1.58–6.61; *p* < 0.01), a history of cancer (OR, 2.18; 95% CI, 1.13–4.20; *p* < 0.05), and low PhA (OR, 0.69; 95% CI, 0.50–0.95; *p* < 0.05), excluding PBF, were found to be significantly associated with the 3-month mortality.

### 3.3. Phase Angle and Mortality of Patients with HAD

**Figure 3:**
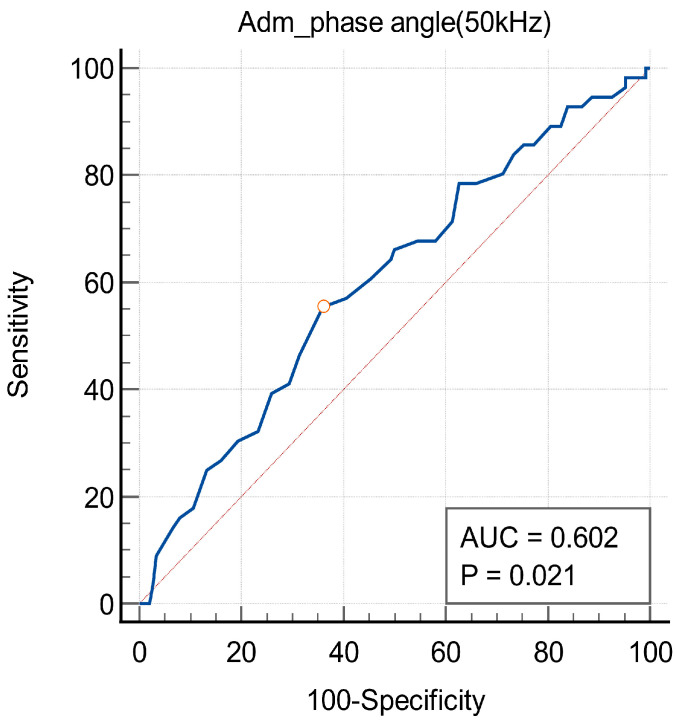
ROC curve of the ability of the phase angle to predict 3-month mortality. The empty circle on the curve indicates the cut-off value. AUC, area under the curve; ROC, receiver operating characteristic.

**Figure 4:**
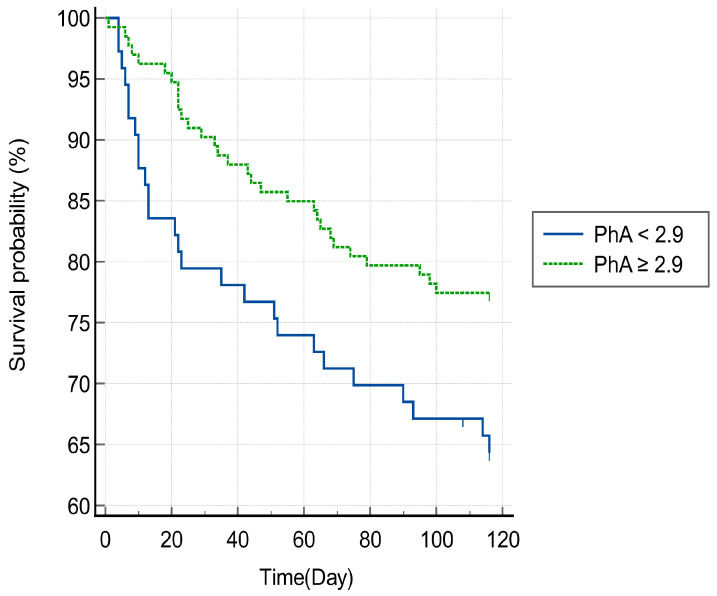
Kaplan–Meier 3-month survival plot for mortality risk of the two groups using cut-offs based on phase angles obtained using bioelectrical impedance analysis (log-rank test: *p* < 0.05). PhA, phase angle.

**Figure 5:**
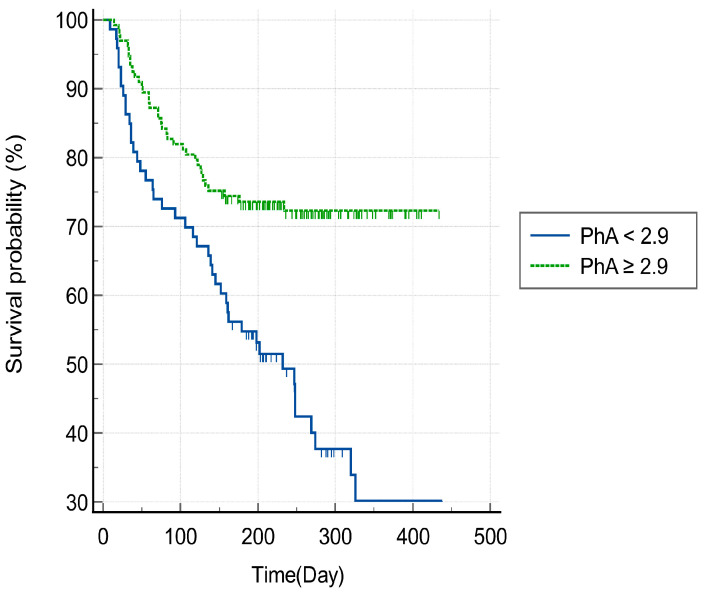
Kaplan–Meier survival plot for mortality risk of the two groups using cut-offs based on phase angles obtained using bioelectrical impedance analysis (log-rank test: *p* < 0.0001). PhA, phase angle.

In the ROC curve analysis, the best cut-off value of the PhA for predicting 3-month mortality was 2.9° (sensitivity = 55%, specificity = 64%, area under the curve = 0.60, *p* < 0.05) (Figure 3). Figure 4 shows a Kaplan–Meier survival curve which confirms mortality within 3 months after HAD diagnosis based on the PhA cut-off value and shows that a PhA of less than 2.9° is associated with a statistically significant increase in 3-month mortality (log-rank test; *p* < 0.05). In addition, Figure 5 shows a Kaplan–Meier survival curve that displays the mortality at an average of 7 months after HAD diagnosis, using the same PhA cut-off value. The results show that when the PhA is below 2.9°, a significant increase is observed in the mortality at 7 months (log-rank test; *p* < 0.0001).

## 4. Discussion

This is the first study of mortality and associated risk factors in patients with HAD following the implementation of an automated screening system for those at risk of developing HAD. Among patients diagnosed with HAD, 27.1% died within 3 months, and 35.9% died within 7 months. The analysis showed that mortality within 3 months after the diagnosis of HAD was associated with male sex, a history of cancer, and a low PhA. There was also a significant difference in the trend of mortality in patients with HAD when the PhA cut-off point was set at 2.9°.

The mortality rate for patients with HAD in this study was higher than in previous studies. In previous studies of patients with HAD, the 3-month mortality rate was lower than in our study (11% in patients aged ≥70 years) [5], and the 6-month and 1-year mortality rates were similar to the 3-month rate in our study, at 29% and 33%, respectively [33,34]. Additionally, the mortality rate of patients with HAD in this study was higher than the 3-month mortality rate of 17.2% for all patient groups discharged from tertiary hospitals, regardless of HAD diagnosis [35], and higher than the mortality rate observed in studies of a non-HAD group [15]. There are two possible reasons for the higher mortality rate. Firstly, this study may have included more severely ill patients than previous studies. Other studies recruited patients from several hospitals, including geriatric care and community teaching hospitals as well as tertiary hospitals, and excluded patients admitted to ICUs or targeted patients at Geriatric Post-Acute Care Units [34,36]. Secondly, the composition of the included patients’ diagnoses differed from that of other studies, which could have contributed to the difference in mortality rates. A previous study of patients admitted to the post-acute care unit divided patients into several disease groups and compared the mortality rate; the deconditioning patient group had significantly higher 1-year mortality than the stroke or orthopedic fracture group [36]. The mortality rate may have been higher in this study because it excluded patients with conditions like fractures or strokes and included only patients who were primarily deconditioned.

In this study, a history of cancer and male sex were significantly associated with 3-month mortality in patients with HAD. First, cancer is the leading cause of death in South Korea and is known to increase the severity of hospitalized patients [37]. Notably, previous studies have reported that older hospitalized patients with a history of cancer have significantly higher mortality rates regardless of the initial diagnosis [38]. It is also known that patients with cancer are more likely to develop new disabilities during hospitalization, and the resulting decline in functional status is associated with mortality in patients with cancer after discharge [16]. Second, in this study’s subgroup analysis on sex, males had a significantly higher rate of sarcopenia than females when classified according to the criteria of the AWGS (*p* < 0.05) and a higher rate of having COPD as an underlying disease (*p* < 0.01). Because skeletal muscle mass is associated with immune mechanisms and glucose and protein metabolism [39,40], a decrease in skeletal muscle mass is known to affect physiologic function adversely, and our results are consistent with previous reports that a low SMI is associated with higher mortality in critically ill patients [41,42,43]. Furthermore, COPD is one of the leading causes of mortality worldwide and has been reported to increase mortality in hospitalized patients with COPD due to increased risk of hypertension, diabetes mellitus, and coronary artery disease [44,45,46]. In particular, it is well known that acute exacerbations of COPD can contribute to increased mortality in older adult patients in tertiary care hospitals [47,48,49].

In this study, the PhA was associated with a higher risk of mortality in patients with HAD. PhA assessment via BIA is a cost-effective and straightforward method, making it a valuable prognostic factor in clinical settings [50]. Previous studies have reported that low PhAs, indicative of decreased cellular integrity, are associated with higher mortality across various diseases. For example, PhA thresholds for increased mortality have been reported to range from <4.4° to <5.8° for patients with cancer, <5.4° for cirrhosis, <4.2° for heart disease, and <3.5° for older adults [17,18,19,31,50,51]. However, the PhA has received less attention in hospitalized patients, and research in this area is limited. Notably, the PhA cut-off identified in this study was lower than those documented in other studies involving critically ill patients [21,52,53]. As noted, the PhA cut-offs for elevated mortality risk vary significantly between diseases and even within the same condition, likely due to differences in patient demographics. Although the PhA cut-off in this study was relatively low compared to other studies, the findings are consistent with previous research on critically ill older patients (men ≤3.29°, women ≤3.01°) [20].

This study had some limitations. First, it is challenging to generalize the results of this study because it only included Asian patients admitted to a single tertiary hospital. This limitation is planned to be addressed through future multi-center research. Second, this study focused only on patients diagnosed with HAD among those admitted to a tertiary hospital, and therefore caution should be taken when generalizing the results. Third, as this study is a retrospective study, it was not possible to assess the functional status of patients before and after hospitalization using the same methods, and thus the degree of functional decline could not be quantitatively presented. Lastly, this study assessed HAD in patients referred through an automated screening system rather than including all hospitalized patients. Therefore, some patients may not have been included in the analysis. Nevertheless, this study is valuable because it is the first to use patient information from an HIS to screen patients at risk for HAD. The findings show that body composition factors, such as PhAs, are associated with mortality in patients with HAD. Additionally, we defined patients with HAD as those who were previously independent in their daily activities but experienced a new functional decline after hospitalization. This definition can aid in targeting these individuals for intervention and rehabilitation planning [54].

## 5. Conclusions

In conclusion, patients with HAD had a high mortality rate, with male sex and a history of cancer identified as the main risk factors. Moreover, low PhAs were associated with increased short-term mortality in patients with HAD. The findings from this study could facilitate the early identification of patients with HAD in clinical settings, thereby enhancing their prognoses.

## Figures and Tables

**Table 1:** Characteristics of patients with HAD.

**Variable**	**Total Population,** **n = 206 (100%)**	**3 Months** **Survivors,** **n = 150 (72.8%)**	**3 Months** **Non-Survivors,** **n = 56 (27.2%)**	***p*-Value**
Age, mean (SD)	73.1 (12.5)	73.2 (12.6)	72.8 (12.4)	0.85
Sex (Male, %)	115 (55.8)	73 (48.7)	42 (75.0)	<0.001
BMI, mean (SD)	21.4 (3.9)	21.2 (3.9)	21.8 (4.1)	0.40
Former/current smoker (%)	65 (31.6)	42 (28.0)	23 (41.1)	0.07
Comorbidities				
Hypertension (%)	108 (52.4)	80 (53.3)	28 (50.0)	0.67
Diabetes mellitus (%)	93 (45.2)	71 (47.3)	22 (39.3)	0.30
Chronic kidney disease (%)	43 (20.9)	31 (20.7)	12 (21.4)	0.90
Coronary artery disease (%)	32 (15.5)	24 (16.0)	8 (14.3)	0.76
Heart failure (%)	20 (9.7)	15 (10.0)	5 (8.9)	0.82
Cerebrovascular disease (%)	33 (16.0)	23 (15.3)	10 (17.9)	0.66
Asthma (%)	9 (4.4)	7 (4.7)	2 (3.6)	1.00
COPD (%)	12 (5.8)	9 (6.0)	3 (5.4)	1.00
Cancer (%)	73 (35.4)	44 (29.3)	29 (51.8)	<0.01
History of ICU admission (%)	44 (21.4)	37 (24.7)	7 (12.5)	0.06
Total ICU days, mean (SD) ^(a)^	11.6 (9.3)	11.8 (9.7)	10.4 (7.0)	0.72
Physical function				
MMT, mean (SD)	39.2 (7.6)	39.5 (6.6)	38.2 (9.6)	0.36
BBS, mean (SD)	7.2 (9.9)	7.9 (10.4)	5.6 (8.5)	0.14
Grip ^(b)^ (Low, %)	200 (97.1)	145 (96.7)	55 (96.7)	1.00
Activities of daily living				
MBI, mean (SD)	26.7 (24.3)	28.6 (23.8)	21.5 (25.1)	0.07
Body composition				
SMI ^(c)^ (Low, %)	199 (79.3)	159 (77.2)	40 (71.4)	0.23
Phase angle, mean (SD)	3.4 (1.2)	3.5 (1.2)	3.1 (1.1)	<0.05
PBF (%), mean (SD)	28.1 (11.2)	29.1 (10.9)	25.4 (11.4)	<0.05

Values are presented as mean ± SD or number (%); HAD, hospital-associated disability; SD, standard deviation; BMI, body mass index; COPD, chronic obstructive pulmonary disease; ICU, intensive care unit; MMT, manual muscle test; BBS, Berg Balance Scale; MBI, modified Barthel Index; SMI, skeletal muscle mass index; PBF, percentage body fat. ^(a)^ Analysis only included patients admitted to the ICU; ^(b)^ analysis was performed by segregating data based on the 2019 AWGS guidelines for normal handgrip strength values (≥28.0 kg for males and ≥18.0 kg for females). Low handgrip strength was coded as 1, and normal as 0. ^(c)^ Analysis was performed by segregating data based on the 2019 AWGS guidelines for normal SMI values (≥7.0 kg/m^2^ for males and ≥5.7 kg/m^2^ for females). Low muscle mass was coded as 1, and normal as 0.

**Table 2:** Univariate and multivariate analysis of factors affecting 3-month mortality.

	**Univariate**			**Multivariate**		
**Variables**	**OR**	**95% CI**	***p*** ***-*** **Value**	**OR**	**95% CI**	***p*** ***-*** **Value**
Sex (male)	3.16	1.60–6.27	<0.01	3.23	1.58–6.61	<0.01
Cancer	2.59	1.38–4.86	<0.01	2.18	1.13–4.20	<0.05
Phase angle (50 kHz)	0.74	0.54–0.99	<0.05	0.69	0.50–0.95	<0.05
PBF (%)	0.97	0.94–0.99	<0.05			

OR, odds ratio; CI, confidence interval; PBF, percentage body fat.

## Data Availability

The original contributions presented in the study are included in the article, further inquiries can be directed to the corresponding author.
